# Attendance of Students at Medical Schools

**Published:** 1863-12

**Authors:** 


### EDITORIAL DEPARTMENT.

### ATTENDANCE OF STUDENTS AT MEDICAL SCHOOLS.

We notice by our exchanges that most of our Medical Colleges are favored by greatly increased numbers of Students. This is due, in some degree, to the great demand for medical men in the army, and the readiness with which the young men of the profession find remunerative employment.

It is sometimes objected to by the army Surgeons that they are young and inexperienced. This is true in good degree; all the active young men, not fully employed in private practice, at once turned their attention to the army, and found a ready opening; and others who had given attention to various callings, to the neglect of professional pursuits, have also obtained place and commenced anew the practice of Medicine. The *young* Surgeons have proved most valuable to the service in many respects. It has been found that those advanced to over forty years of age have not often been able to endure the hardships of military life; while young men, fresh from College, have rarely failed to make efficient and capable medical officers.

The army is offering to recent graduates in medicine opportunities worthy their attention, and we have no doubt that many of the graduates of the approaching commencements in the Medical Colleges will obtain pleasant positions in the army. Provision has been made, and especial pains taken that young men who graduate, shall be fully instructed in military practice, so that it is probable that those who hereafter enter the army will be better qualified to enter at once upon the duties of the service.—This great increase of attendance is suggestive of many things both to the Students themselves as well as to the Boards of Instruction. To the former it indicates that though the field is large, they must enter heartily and energetically in the strife for excellence or be lost in obscurity; since active competition will meet them in every pathway of professional ambition.— The latter might perhaps regard it as a favorable time to introduce improvements in their systems of instruction, which have long been urged upon them and regarded important by distinguished teachers and members of the profession.

Reforms in Medical education have been suggested and urged for a series of years. The American Medical Association was mainly organized for the promotion of this object, and something has been accomplished through its agency. Since its organization most Medical Colleges have attempted reforms; have extended their lecture terms, increased the number of their Professorships, instituted daily examinations of their classes, incorporated Hospital Clinical instructions, both in Medicine and in Surgery. made more ample provisions for teaching Physiology, Pathological Anatomy, Pharmacy, Medical Jurisprudence, &c. Notwithstanding this has been accomplished, there are remaining improvements which can hardly be said to have been attempted.

We have not yet heard from the Medical College where preliminary examinations are made and thorough English education insisted upon; though we have seen in the annual announcements that the terms of graduation are, "twenty-one years of age; three years study with a regular practitioner of medicine, and an adequate knowledge of the *Latin* language; attendance upon two full courses of Medical Lectures, the last at this Institution, and satisfactory examination before the Faculty and Curators."

There are other improvements which have been long urged, but not attempted by our schools. Possibly the time when such advancements as have been suggested, could be safely inaugurated, has never appeared; we are not disposed to presume it ever has, since the attempt has not been made, though heartily desired. A great deal of priority and superiority in reforms, are claimed in introductory addresses before freshman classes and friends of favorite institutions, but after all, a vast amount yet remains to be accomplished, even granting all that we have seen claimed.

Buffalo College has published nothing in this line of "achievements," and we presume it would not be willing to have us speak for it; but if we do not say Buffalo College, and place under it whatever we think has any connection with it, some one may think we *mean* Buffalo while we avoid *saying* anything about it.

If our friends knew us pretty well, they would understand that we never mean what we do not say, and that we propose to say, in such matters, what we mean and honestly believe to be true.

The Buffalo Medical College, has gradually increased in all the elements of true prosperity, and has never seen a time in its history when it had greater reason for congratulation than the present; never a time when it could so well afford to maintain becoming modesty. It has no occasion for boasting of its success, and we presume is sensible that the summit of excellence is not yet fully attained. While it has been forward in originating and early in adopting every improvement, it is probably conscious of defects, and looking forward to the time when a higher standard of attainment shall be reached.

Young men who hold a Diploma from its Faculty possess good evidence of thorough primary qualification in all the branches of medical knowledge; while opportunity has not been wanting for them to become practically familiar with the practice of Medicine and Surgery. We hope and expect to see the time when the standard of admittance and graduation in the University of Buffalo will not only equal, but excel, that of sister institutions.

Young men who are to commence the study of medicine should possess at least an adequate knowledge of the *English* language; but this pre-requisite we are sorry to say is not insisted upon. The archives of all our medical schools furnish evidences of this, and of the importance of more thorough preliminary education.

We do not mean to say that there are not physicians who, with limited early opportunities, have attained to distinction in the profession, and far out-done many who were favored with classical culture; but they were men far above the average, in natural strength and vigor of intellect, and have pursued their objects with a devotion and perseverance to be found only with few among the many; they have overcome obstacles and surmounted difficulties which would have overwhelmed and discouraged less resolute and determined men.

While suggesting this topic for the consideration of those interested, we would yet say that we believe the occasion for reform is gradually lessening. Medical students are better educated before entering the schools than formerly; they are seeing more and more the importance of thorough qualification both previous to, and after commencing their professional course of study; they are devoting themselves more exclusively to professional attainment, and making their period of pupilage more productive of positive knowledge. They do not, as formerly, look forward to years of waiting for occasion to put in practice what they have learned, but expect to be immediately employed in active labor. A more favorable time to enter the profession of Medicine was never known; and we are glad to believe that more thoroughly qualified, energetic and capable young men were never seeking to enter it.

